# Serum and Vitreous Levels of Placenta Growth Factor in Diabetic Retinopathy Patients: Correlation With Disease Severity and Optical Coherence Tomographic Parameters

**DOI:** 10.7759/cureus.54862

**Published:** 2024-02-25

**Authors:** Joana Mesquita, Fátima Milhano Santos, João Paulo Sousa, Sara Vaz-Pereira, Paulo Tavares-Ratado, Arminda Neves, Rita Mesquita, Cândida Teixeira Tomaz

**Affiliations:** 1 Pharmacy, Centro de Investigação em Ciências da Saúde, Universidade da Beira Interior (CICS-UBI), Covilhã, PRT; 2 Biochemistry, Instituto de Investigación Sanitaria de la Fundación Jiménez Díaz, Madrid, ESP; 3 Ophthalmology, Centro Hospitalar de Leiria, Leiria, PRT; 4 Ophthalmology, Hospital de Santa Maria, Lisbon, PRT; 5 Clinical Research, Medical Sciences, Universidade da Beira Interior, Covilhã, PRT; 6 Medicine, Faculty of Medicine, Universidade de Lisboa, Lisbon, PRT; 7 Pharmacology and Therapeutics, Centro de Investigação em Ciências da Saúde, Universidade da Beira Interior (CICS-UBI), Covilhã, PRT

**Keywords:** placenta growth factor (plgf), novel therapeutic approaches, vitreous humor, vascular endothelial growth factor, diabetic retinopathy

## Abstract

Purpose

The primary objective of this study was to compare placenta growth factor (PlGF) levels in the serum and vitreous of diabetic retinopathy (DR) patients to non-diabetic controls. Additionally, the study aimed to establish associations between serum and vitreous PlGF concentrations and to examine the correlation between vitreous PlGF in DR patients and morphological parameters.

Methods

This study included serum and vitreous samples from 38 patients, including 21 patients with DR and 17 non-diabetic controls. The control group included non-diabetic patients with rhegmatogenous retinal detachment with retinal tears secondary to posterior vitreous detachment or trauma. PlGF levels were quantified in vitreous and serum samples using an enzyme-linked immunosorbent assay (ELISA). Optical coherence tomography (OCT) scans from DR patients were evaluated to measure the central retinal thickness (CRT) and macular volume (MV).

Results

DR patients had significantly higher mean vitreous PlGF levels compared to non-DR patients (70.0±39.2 vs. 46.47±9.7 pg/mL, p-value=0.004). However, no significant increase in mean serum PlGF levels was observed in DR patients (p-value=0.232). Within the DR group, proliferative DR (PDR) patients presented significantly higher vitreous PlGF levels than non-PDR (NPDR) patients (76.5±41.0 vs. 42.5±5.0 pg/mL, p-value=0.009). There was no association between serum and vitreous PlGF levels. The correlation between vitreous PlGF levels and morphological parameters was r_sp_=0.175, p-value=0.488 for CRT, and r_sp_=0.288, p-value=0.262 for MV.

Conclusion

This study emphasizes the important role of PlGF in neovascularization, specifically highlighting its overexpression exclusively in vitreous from PDR patients. The observed increase in PlGF levels may be indicative of disease severity. The lack of correlation between vitreous and serum PlGF levels suggests a potential dissociation between intravitreal and systemic PlGF synthesis. Consequently, targeting PlGF in therapeutic approaches may offer an additional strategy for ocular pathologies with a neovascular component.

## Introduction

The incidence of diabetes is increasing every year. Diabetic retinopathy (DR), a microvascular complication and a major cause of blindness among the working-age population, is a common condition associated with the onset of diabetes [1]. Clinically, DR is classified as either non-proliferative DR (NPDR) or proliferative DR (PDR). NPDR presents microaneurysms, retinal hemorrhages, hard exudates, cotton wool spots, venous beading, and intra-retinal microvascular abnormalities. In contrast, the hallmark of PDR is the presence of retinal neovascularization driven by retinal ischemia, hypoxia, and vascular endothelial growth factor A (VEGF-A) stimulation. The new blood vessels proliferate, leak, and may lead to vitreous hemorrhage, tractional detachment, or neovascular glaucoma, resulting in irreversible visual loss [2]. VEGF-A is the most studied and influential molecule in the DR process [3]. Therefore, the use of anti-VEGF therapies has been a significant improvement in the treatment of ocular diabetic disease [4]. However, disease recurrence is common among these patients. While anti-VEGF therapies seem to be generally safe, the long-term consequences remain uncertain [4]. Given the increasing incidence of diabetes mellitus and, consequently DR, coupled with the substantial burden associated with current DR treatments, it becomes imperative to shift attention toward other contributory molecules and the development of novel targeted therapies to fight vision loss [1,5].

Besides VEGF-A, other growth factors, such as the placenta growth factor (PlGF), seem to be implied in this process [3]. The PlGF was discovered after VEGF-A, and it was considered the second member of the VEGF family [3]. Its alternative splicing generates four isoforms (PlGF-1, PlGF-2, PlGF-3, and PlGF-4). PlGF binds to vascular endothelial growth factor receptor 1 (VEGFR-1), soluble FMS-like tyrosine kinase-1, and neuropilins-1 and -2 [6,7]. The PlGF binds specifically to VEGFR-1; however, it may activate vascular endothelial growth factor receptor 2 (VEGFR-2) through indirect mechanisms. PlGF may bind VEGFR-1, dislocating and freeing VEGF-A, thus increasing its availability to bind and activate VEGFR-2 [7]. PlGF and VEGF-A may also produce heterodimers capable of binding and activating VEGFR-1 [8].

PlGF appears to play a significant role in pathologies involving ischemia, malignancy, inflammation, and enhanced vascularization. In fact, the role of PlGF has been observed in pathological states rather than in physiological states. Several studies showed that PlGF has a negligible role during normal vascular development and maintenance, but it is essential for the angiogenic and inflammatory switch in some diseases [8].

Pharmacological studies focused on loss-of-function and gain-of-function led to the characterization and identification of therapeutic needs in PlGF delivery and blockage [6]. Some pathological conditions may improve due to delivering of PlGF. In the cardiovascular system, PlGF preserves cardiac performance after infarction by inducing revascularization of ischemic myocardium and vessel enlargement, thus playing a significant role in myocardial angiogenesis, regulation of vascular growth in pathological states, and a selective action in modulating pathological rather than physiological vascular development. In the central nervous system, PlGF is upregulated in neurons and vascular cells during cerebral ischemia, having neuroprotective properties. PlGF is also overexpressed in the skin during wound healing. Therefore, increased PlGF levels lead to an increase in angiogenesis, thus improving wound healing and ultimately skin regeneration. Additionally, during bone fracture repair, colitis, sepsis, and preeclampsia, where healing angiogenesis is present, PlGF therapy delivery may help restore normal functions [6].

While the upregulation of PlGF proved to be beneficial in certain pathological conditions, it exacerbates others, such as ocular diseases, by promoting neovascularization [9]. PlGF deficiency or PlGF receptor neutralization in animal models was shown to decrease choroidal neovascularization. Additionally, the intraocular delivery of PlGF has been demonstrated to contribute to the progression of DR. The pharmacological inhibition of PlGF hinders neovascularization by suppressing vessel abnormalization and vascular leakage while enhancing VEGF-targeted inhibition and mitigating ocular inflammation [10]. The role of PlGF has been a subject of controversy in the literature. However, recent studies have unequivocally established its involvement, not only in angiogenesis but also in inflammatory modulation [3,8]. According to Huo et al. [11], the impact of PlGF on choroidal neovascularization is case-dependent, through a mechanism of co-inhibition where PlGF reinforces the effect of anti-VEGF-A inhibition [11].

Considering its role in DR, serum and vitreous PlGF levels were evaluated by enzyme-linked immunosorbent assay (ELISA) in DR patients and compared to a non-diabetic control group (with rhegmatogenous retinal detachment). The research aimed to establish associations between PlGF levels in vitreous and serum, as well as to associate these levels with disease severity, and structural parameters, such as central retinal thickness (CRT) and macular volume (MV), performed by optical coherence tomography (OCT). A better understanding of the expression and behavior of this molecule in eye diseases and its correlation with functional and structural outcomes will contribute to the development of better-targeted therapies.

## Materials and methods

Participants and study design

This study was conducted following the Declaration of Helsinki and approved by the Institutional Review Board, Ethics Committee for Health of Centro Hospitalar de Leiria (reference - CHL-15481). Informed consent was obtained from all subjects involved in the study. Undiluted samples of vitreous humor and serum were collected from patients who were submitted to pars plana vitrectomy (PPV) due to different ocular pathologies. Samples from patients were selected for analysis and included for PlGF quantification if they met all of the following inclusion criteria: (1) sufficient sample volume collected to allow the confirmation of the results through repeated ELISA tests; (2) patients with a confirmed diagnosis of DR; (3) patients who were last treated for their eye condition (with anti-VEGF, corticosteroid, or laser) more than three months before PPV surgery; and (4) Naïve patients to aflibercept, either systemically or intravitreally. Concerning exclusion criteria, we have excluded the following from the DR and the control group: (1) all patients with diseases that may confound the results, such as cancer, inflammatory diseases, and autoimmune diseases; (2) hemolyzed samples; (3) any additional systemic metabolic disease or intravitreal or systemic inflammation; and (4) patients who received medications that potentially could affect the results, including drugs that bind to PlGF (such as aflibercept, brolucizumab, and faricimab). Both type 1 and type 2 diabetic patients were enrolled in the study. Diabetic patients underwent PPV because of vitreomacular interface alterations or proliferating fibrovascular membranes and retinal traction, or tractional retinal detachment, or when previous laser photocoagulation (focal, grid, or panretinal) or pharmacologic intervention alternatives failed or were not possible.

Rhegmatogenous retinal detachment patients with retinal tears secondary to posterior vitreous detachment or trauma and with no reports of other eye diseases or disorders that may confound the results were selected to serve as a control sample, minimizing the bias caused in the interpretation of the results. In addition, the clinical history of each patient was reviewed to confirm the patient's diagnosis, baseline characteristics, and any concomitant medications or associated diseases. All information regarding additional drugs used to treat these patients' eye disorders, even if performed three months before the vitrectomy, was gathered. At the end of the selection, a total of 38 patients were included: 21 with DR (12 female and nine male patients) and 17 with rhegmatogenous retinal detachment (five female and 12 male patients). For the correlation of vitreous PLGF levels with the CRT and MV, only patients with DR and complete data were included in this analysis. Only one eye from each patient was studied.

Collection of samples from patients

Undiluted vitreous humor and serum samples were collected from PPV patients at a public hospital (Centro Hospitalar de Leiria, Leiria, Portugal). Just before the surgery, serum samples were collected in an appropriate serum sterile tube. For the serum preparation, after the whole blood collection (about 4 mL), the blood was allowed to clot by placing it in a resting position at room temperature for 30 minutes. The clotted material was removed by centrifuging the sample for 10 minutes at 1,000-2,000 x g in a refrigerated centrifuge. The serum was collected in an appropriate sterile tube and frozen at -80°C until further analysis. Vitreous humor was collected at the beginning of the PPV (core vitrectomy). The vitrectomy tubes were detached and attached to a syringe (in coordination with vitrectomy aspiration at the beginning of the surgery). Before turning on the intravitreal infusion, an undiluted sample of vitreous was obtained by aspiration into a 2 mL syringe attached to the vitreous cutter. The volume of undiluted vitreous collected will be the maximum amount that the surgeon can collect without posing any risks to the patient. Vitreous samples transferred to sterilized tubes were immediately placed on dry ice until stored at -80°C for further analysis. To minimize sample degradation, all sample preparation procedures, whether serum or vitreous humor, were carried out in a room attached to the surgery room.

Measurement of vitreous and serum PlGF levels

Quantification of vitreous and serum PlGF levels was performed by the ELISA kit for human samples (ABIN1379954, Assay Biotechnology, San Francisco, CA), according to the protocol specified by the manufacturer. A volume of 100 µL of vitreous or plasma was used to perform the ELISA test. The detection range was between 32 and 2000 pg/mL, and the sensitivity or the minimum detectable level was less than 32 pg/mL.

Quantitative analysis of OCT

The OCTs, performed before the surgery for all DR patients, were evaluated to measure CRT (μm) and MV (mm^3^) through the interpretation of the macular map. The OCTs were performed in s Spectralis OCT (Heidelberg Engineering, Heidelberg, Germany) for the duration of the study. The software of the Heidelberg Engineering OCT is based on the Heidelberg Eye Explorer: HEYEX1.

Statistical analysis

For statistical analysis, patients were categorized into six distinct groups: DR versus non-DR, PDR versus NPDR, and DME versus non-DME. Statistical Product and Service Solutions (SPSS, v22.0.; IBM SPSS Statistics for Windows, Armonk, NY) was used for statistical analysis. Because the variables did not have a normal distribution, the Mann-Whitney test was utilized (analyzed with the Shapiro-Wilk test). To accept or reject the null hypothesis, a level of significance was set to (α) ≤ 0.05. The median, interquartile range, and minimum and maximum values of the samples were also calculated. The correlation between the quantitative variables was analyzed using Spearman's ordinal correlation coefficient. The sample power was calculated using the G*Power software (version 3.9.7; The G*Power Team, Germany) through a unilateral test with a mean effect size and an alpha of 0.05. The two groups to be compared, having sizes of 21 and 17, provided a power test of 0.329. The results were visualized in graphs obtained with GraphPad Prism (version 9.0; GraphPad Software, San Diego, CA).

## Results

Study population

This study included serum and vitreous samples from 38 patients. Concerning the baseline characteristics, the mean age between the diabetic (n=21) and control group (n=17) was 60.00±25.35 and 68.65±9.69 years, respectively. Of the total of 21 DR patients, 17 (80.9%) had PDR, and the remaining four diabetic patients (19.1%) had NPDR. Table 1 summarizes the demographic and clinical characteristics of the study population, as well as the concomitant drugs and non-drug therapy used by patients three months before vitrectomy.

**Table 1:** Baseline demographic and clinical characteristics of the selected study subjects. Previous treatment therapies were performed up to three months before vitrectomy. N.A. - Not applicable; DME - Diabetic macular edema; NPDR - Non-proliferative diabetic retinopathy; PDR - Proliferative diabetic retinopathy; PPV - Pars plana vitrectomy; SD - Standard deviation

	DR patients (including PDR and NPDR)	Non-DR patients (control group)
Sample size (%)	21 (55.3%)	17 (44.7%)
Sex - male (n, %)	11 (52.4%)	12 (70.0%)
Sex in PDR patients - male (n, %)	8 (47.1%)	
Sex in NPDR patients - male (n, %)	1 (75.0%)	
Mean age (years) ±SD	60±25	69±10
Diabetes type 2 (n, %)	21 (100%)	Not applicable
Other characteristics of DR patients		
PDR patients % (n)	80.9% (17)	N.A.
NPDR patients % (n)	19.1% (4)	N.A.
DME % (n) Overall in DR patients	71.4% (15)	N.A.
DME % (n) in PDR patients	82.0% (14)	N.A.
DME % (n) in NPDR patients	25% (1)	N.A.
Indications for PPV for DR patients		
PDR	80.9% (17)	N.A
Vitreomacular traction (NPDR)	19.1% (4)	N.A
Indications for PPV for non-DR patients (control group)		
RRD with retinal tears secondary to posterior vitreous detachment	N.A	94.1% (16)
RRD with retinal tears secondary to trauma	N.A	5.9% (1)
Previous treatments for diabetic ocular disease performed up to 3 months before PPV/sample collection		
Laser % (n)	100% (21)	N.A.
Ranibizumab % (n)	14.3% (3)	N.A.
Triamcinolone acetonide % (n)	14.3% (3)	N.A.

Comparison of vitreous and serum PlGF levels between the DR and control groups

The results of the statistical analysis, including calculations of the mean, median, standard deviation, interquartile range, and minimum and maximum values, are presented in Table 2.

**Table 2:** Descriptive statistics for PlGF vitreous and serum concentration (pg/mL) in the groups of DR and non-DR patients. DR - Diabetic retinopathy; Non-DR - Non-diabetic retinopathy; PlGF - Placenta growth factor; SD - Standard deviation *Statistically significant results

	PlGF vitreous concentration (pg/mL)		PlGF serum concentration (pg/mL)	
	DR patients (n=21)	Non-DR patients (n=17)	DR patients (n=21)	Non-DR patients (n=17)
Mean	70.00 *	46.47 *	50.48	48.82
Median	50.00 *	40.00 *	50.00	50.00
SD	39.24	9.96	2.18	6.00
Minimum	40 *	40 *	50	40
Maximum	210 *	80 *	60	60
Interquartile range	35 *	10 *	0	5

**Figure 1:**
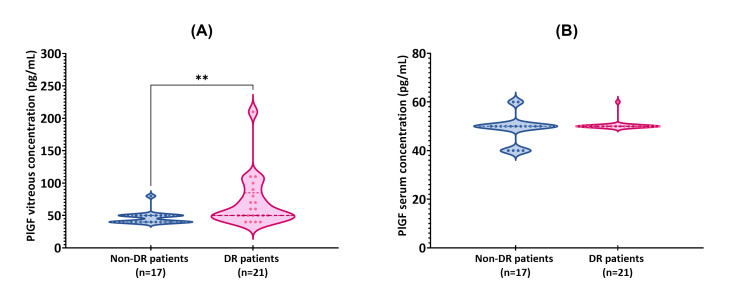
Comparison of the placenta growth factor (PlGF) levels in a) vitreous and b) serum between patients with diabetic retinopathy (DR) (n=21) and non-diabetic control (non-DR) (n=17). The Mann-Whitney test was utilized to determine the statistically significant difference between the two groups. The median, interquartile range, and minimum and maximum values are represented in the violin plots. a) ** p-value=0.004. b) The results were not statistically significant: p-value=0.232.

Vitreous samples of DR patients had significantly higher concentration values of PlGF in comparison with non-diabetic patients, with a mean of 70.00±39.24 pg/mL vs. 46.47±9.96 pg/mL, respectively (Z=-2.847, p-value=0.004). The results of median, interquartile range, and minimum and maximum values can be seen in Figure 1A.

Concerning the serum samples, PlGF was not found to be statistically increased in DR patients, with a mean of 50.50±2.18 pg/mL in DR patients vs. 48.80±6.0 pg/mL in non-DR patients (Z=-1.196, p-value=0.232) (Figures 1B). A median of 50.0 for both groups was observed, as illustrated in Figure 1B.

Comparison of vitreous PlGF levels between patients with PDR and NPDR

**Figure 2:**
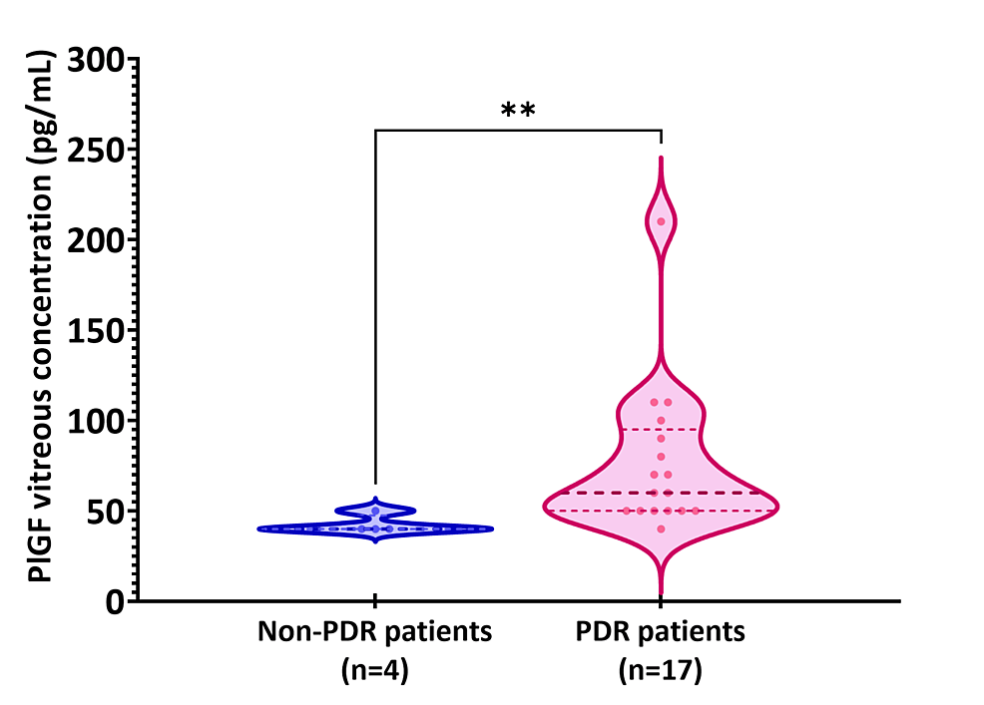
Comparison of the placenta growth factor (PlGF) levels in the vitreous between patients with proliferative diabetic retinopathy (PDR) (n=17) and non-proliferative diabetic retinopathy (NPDR) (n=4). The Mann-Whitney test was utilized to determine the statistically significant difference between the two groups. The median, interquartile range, and minimum and maximum values are represented in the violin plots. **p-value=0.009.

In vitreous samples, PlGF concentration was significantly higher in patients with PDR than patients with NPDR: 76.5±41.0 pg/mL vs. 42.5±5.0 pg/mL (Z=-2.612, p-value=0.009), as shown in Figure 2. Despite this interesting finding indicating a link between PlGF vitreous levels and disease severity, we cannot confirm this assumption due to the NPDR group's small sample size.

Comparison of vitreous PlGF levels between DR patients with diabetic macular edema (DME) and without DME

**Figure 3:**
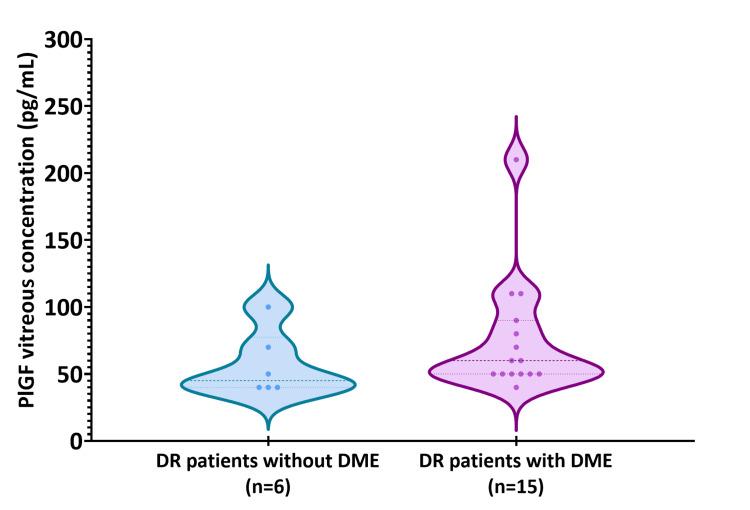
Comparison of the placenta growth factor (PlGF) levels in the vitreous between patients with diabetic retinopathy (DR) with diabetic macular edema (DME) (n=15) and without DME (n=6). The Mann-Whitney test was utilized to determine the statistically significant difference between the two groups (p-value=0.178). The median, interquartile range, and minimum and maximum values are represented in the violin plots.

The comparison of patients with DR with and without DME revealed that patients with DME had higher median and mean PlGF levels. However, it should be noted that these findings were not statistically significant (p-value=0.178), as can be shown in Figure 3. Although the DME group reached the highest PlGF level (210 pg/mL), the obtained minimal values, along with the interquartile range, demonstrated similarity in both groups. Table 3 summarizes the results of the statistical analysis, including the mean, median, standard deviation, interquartile range, and minimum and maximum values.

**Table 3:** Descriptive statistics for the PlGF vitreous concentration (pg/mL) in the group of DR patients with and without DME. DR - Diabetic retinopathy; DME - Diabetic macular edema; PlGF - Placenta growth factor; SD - standard deviation. The results were not significantly significant (p-value=0.178).

	PlGF vitreous concentration (pg/mL)	
	DR patients with DME (n=15)	DR patients without DME (n=6)
Mean	75.33	56.67
Median	60.00	45.00
SD	43.40	24.22
Minimum	40	40
Maximum	210	100
Interquartile range	40	38

Association between vitreous and serum PlGF

**Figure 4:**
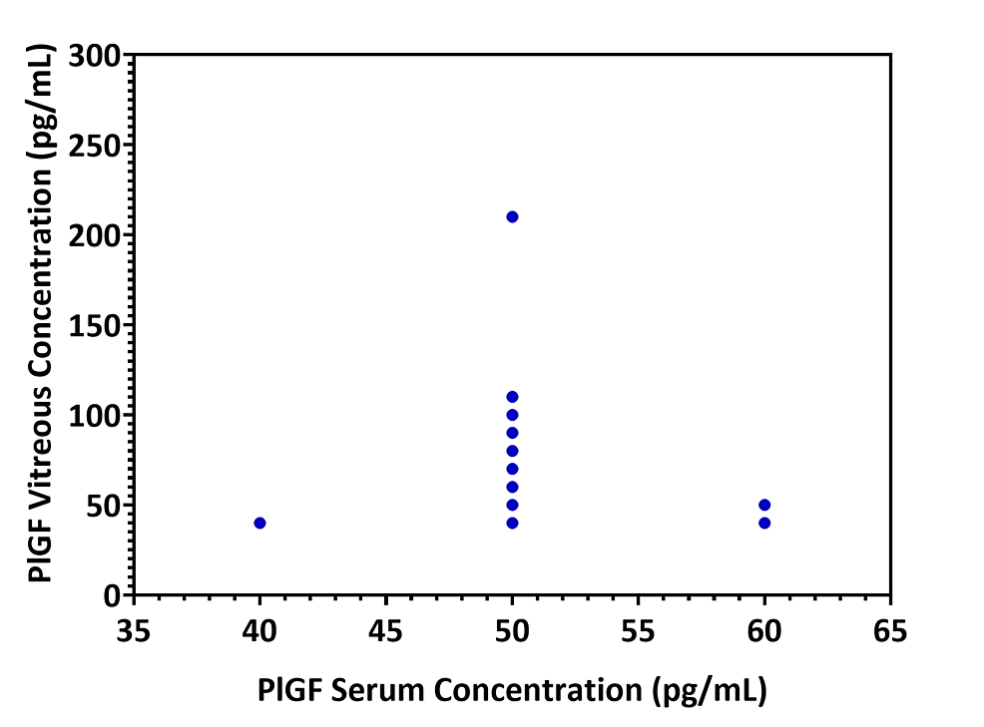
Correlation between the placenta growth factor (PlGF) vitreous and serum levels according to Spearman’s correlation coefficient. The results were not statistically significant (p-value=0.645).

No correlation was found between the concentrations of PlFG in vitreous and serum. The correlation coefficient (rsp=0.077) between vitreous and serum PlGF levels (n=38) was not statistically significant (p-value=0.645) (Figure 4), neither the correlation of PlGF levels between vitreous and serum samples in the DR patient group (n=21) (p-value=0.614; rsp=-0.117).

Association between vitreous and serum levels of PlGF in DR patients and non-DR patients

In the diabetic group (n=21), the correlation between vitreous and serum PlGF levels was not statistically significant and had a correlation coefficient close to zero (r2=-0.113, p-value=0.625). Similarly, the correlation between vitreous and serum PlGF levels was also not statistically significant in the group of non-diabetic patients (n=17), presenting a very low correlation coefficient (r=0.392; p-value=0.119).

Correlation between the vitreous PlGF in DR patients and OCT parameters

**Figure 5:**
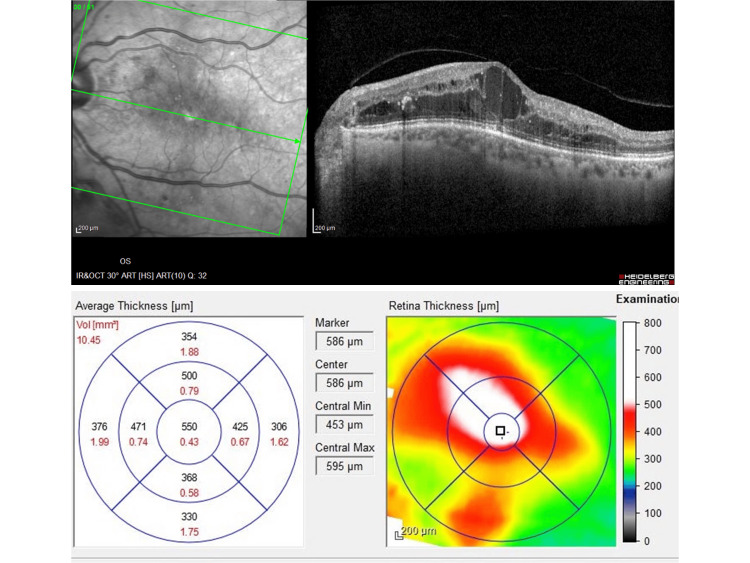
Representative image of an OCT from a DR patient with the measurements of CRT (μm) and MV (mm3) (Heidelberg Engineering, Heidelberg, Germany). Courtesy of Dr. Susana Teixeira

The OCTs of DR patients were analyzed to obtain the values of CRT and MV and correlated them with PlGF intravitreal levels. Figure 5 shows a representative image of the OCT measurements of CRT (μm) and MV (mm^3^) from a DR patient.

**Figure 6:**
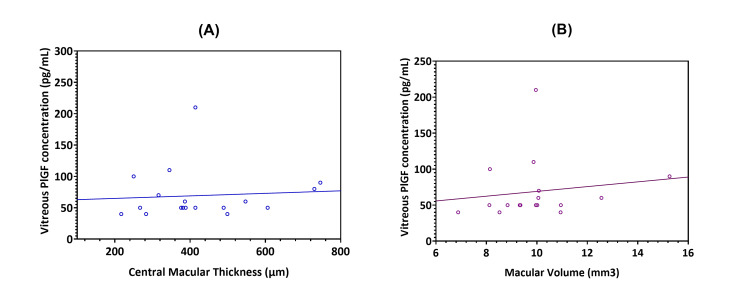
A) Correlation between the placenta growth factor (PlGF) vitreous levels and central retinal thickness (CRT) (µm) in diabetic retinopathy (DR) patients (n=21), analyzed with Spearman’s correlation coefficient (rsp=0.175; p-value=0.488). B) Correlation between the placenta growth factor (PlGF) vitreous levels and macular volume (MV) (mm3) in DR patients (n=21), also analyzed with Spearman’s correlation coefficient (rsp=0.288; p-value=0.262).

The correlation coefficient between vitreous PlGF levels in DR patients and CRT (µm) was moderate (rsp=0.175; p-value=0.488), as shown in Figure 6A. Additionally, the correlation between the MV (mm^3^) and PlGF levels showed a moderate correlation (rsp=0.288; p-value=0.262), suggesting the possibility of a positive relationship between these variables (Figure 6B).

However, the limited sample size of the cohort of diabetic patients (n=21) prevents the capacity to draw meaningful conclusions. As a result, this analysis lacks the statistical power required to reliably detect relationships due to the small sample size of the cohort.

## Discussion

Angiogenesis is controlled by a myriad of pro-angiogenic factors, including not only the members of the VEGF family (VEGF-A, PlGF, VEGF-B, VEGF-C, and VEGF-D) but also epidermal growth factor, transforming growth factor, angiopoietins, fibroblast growth factor, and matrix metalloproteinases. Conversely, anti-angiogenic factors, such as pigment epithelium-derived factor (PEDF), prolactin, angiostatin, vasoinhibins, endostatin, and thrombospondin, counterbalance these pro-angiogenic signals. This intricate interplay of factors contributes to various physiological processes within the human body, with unregulated angiogenesis often serving as a precursor to various diseases [12]. PlGF also plays a significant role in this context, influencing neovascular diseases of the retina. Therefore, studying this growth factor becomes essential for the development of specific therapies, highlighting the potential of PlGF as a strategic target.

In our study, PlGF levels in vitreous and serum were compared between DR and non-diabetic patients. PlGF levels in serum were similar for DR patients and non-diabetic control group, as well as PlGF levels, whether in vitreous or in serum, for non-DR patients. Initially, researchers were exploring the feasibility of utilizing PlGF as a disease marker in serum due to the convenience of testing its levels in this matrix. Bonfiglio et al. [13] conducted a study that validated transforming growth factor β1 (TGFβ1) as a diagnostic and prognostic biomarker in the serum of DR patients. They measured serum levels of VEGF-A, PlGF, and TGFβ1, but only TGFβ1 serum levels predicted disease progression from NPDR to PDR. This supports the hypothesis that TGFβ1 could serve as a biomarker and pharmacological target for DR. However, it also indicates that PlGF serum levels are not useful for predicting DR staging or clinical outcomes [13]. Nevertheless, the results showed no significant changes in PlGF levels in the serum of DR patients, and, therefore, this hypothesis was disregarded. However, this raises questions regarding the origin of PlGF, specifically whether it is primarily synthesized intravitreally. Wu et al. [14] showed that PlGF-1 is expressed in the Muller glial cells and the internal segments of the photoreceptors in the retina. This study revealed that PlGF-1 is significantly upregulated in the retina of diabetic mice during the initial stages of diabetes and remains thereafter. Moreover, elevated concentrations of PlGF-1 can contribute to disrupting the cytokine microenvironment in the retina, resulting in inflammation, and potentially impacting the functioning of retinal cells, including microglia. According to their research, PlGF-1 may have a role in the initial phases of DR, when only microaneurysms are present, and possibly might act in synergy with VEGF to exacerbate damage to the retina [14]. On the other hand, it clarified the fact that PlGF is overexpressed under pathologic conditions.

In contrast, significantly higher levels of vitreous PlGF were observed in DR patients when compared to a control group of non-diabetic patients. Additionally, the comparison of vitreous PlGF levels between DR patients with or without DME revealed a higher concentration in patients with DME, but this difference was not statistically significant. Our findings did not provide sufficient evidence to determine if PlGF has a link with DME, as the majority of DME patients in our study had PDR. These results are according to other studies in the literature, which will be described below, in which high levels of PlGF have been found in the vitreous of patients with DR and other neovascular retinopathies, suggesting a role in the pathogenesis of PDR. Some of these studies and the role of PlGF and its receptor system in DR and other retinal vascular diseases were recently reviewed by Van Bergen et al. [15]. They also emphasized the role of PlGF in neovascularization, vascular leakage, and inflammation, demonstrating the positive effects of PlGF deletion/inhibition on mitigating these key pathological processes in DR and DME.

Mitamura et al. observed significantly higher intravitreous levels of PlGF in active PDR compared to quiescent PDR, suggesting the involvement of this molecule in the early stages of PDR development [16]. Higher PlGF values in DR patients appear to increase with the severity of the disease. Additionally, a significant correlation was found between intravitreous PlGF and VEGF levels in both PDR patients and total subjects. PlGF acts indirectly by potentiating the activity of VEGF in pathological angiogenesis, suggesting the cooperative role of these two molecules in the progression of DR. Therefore, an effective PlGF antagonist would be desirable in all disease stages, but particularly critical in advanced stages, despite the lack of conclusive evidence [16]. Kovacs et al. [17] measured several angiogenic and inflammatory molecules in vitreous collected from patients at different stages of DR and neovascular glaucoma. They found that PlGF was the only protein found to have a statistically significant increase in its levels between each successive stage of DR and neovascular glaucoma, suggesting a correlation with the progression of ischemic retinopathies. Given these findings, the use of anti-PlGF was suggested as an alternative treatment in cases of advanced DR that are at high risk of developing neovascular glaucoma, although further investigation was recommended to assess the efficacy of these drugs [17]. Al Kahtani et al. [18] found that PlGF is significantly downregulated in the vitreous in inactive/quiescent PDR compared to active PDR. Moreover, PlGF levels were correlated with VEGF levels in active PDR. The authors implied the active role of PlGF in PDR due to the strong correlation of PlGF levels with disease progression, highlighting the benefits of PlGF targeting inhibition [18]. Katagiri et al. found that PlGF intravitreal levels were significantly higher in PDR patients with vitreous hemorrhage and fibrovascular proliferative membranes than in NPDR patients. PlGF and VEGF levels were correlated positively, highlighting once again their synergistic effect on the DR progression. However, in this study, the levels of these molecules were also correlated with the levels of leptin, a circulating angiogenic factor that is mainly secreted by adipose tissue [19].

In addition to its angiogenic function, PlGF plays a critical role in the induction of inflammatory responses, acting act as a potent chemoattractant and recruiter of monocytes and macrophages, in ocular and non-ocular diseases (e.g., cancer or pre-eclampsia) [15]. Therefore, despite PlGF's inert function in healthy conditions, its roles in pathological conditions via angiogenic and inflammatory switching mechanisms have been extensively explored. PlGF has been extensively studied in pregnancy and cancer, with the question of whether PlGF can provide an alternative to anti-angiogenic therapy, which encounters issues, such as refractory patients and acquired resistance, a phenomenon that also occurs in the treatment of retinal diseases. Oura et al. performed one of the first studies to report the critical role of PlGF in inducing cutaneous inflammation, in addition angiogenesis, vascular permeability, and edema formation [20]. PlGF-deficient mice displayed a diminished and shortened inflammatory response compared to the wild-type, along with a reduction in inflammatory angiogenesis and edema formation. The synergistic role of PlGF and VEGF in the induction of vascular permeability was also studied, indicating that the vascular permeability induced by PlGF was found to be less potent than VEGF-A alone or VEGF-A/PlGF heterodimers. Nevertheless, studies have indicated that the synergy between PlGF and VEGF-A contributes to pathological angiogenesis. Considering the role of PlGF described in this study, it was suggested that the inhibition of PlGF could be considered as a potential therapeutic approach for this cutaneous condition, acting as an anti-inflammatory drug.

Another interesting result was the lack of association between PlGF levels in vitreous and serum samples, which could suggest a dissociation between the eye and other systems. Another study [14] aimed to examine the correlation between proangiogenic and inflammatory cytokines in vitreous, aqueous, and plasma samples from patients with PDR (n=17) versus controls (n=7). The findings revealed that patients with diabetes exhibited higher levels of vitreous IL-6, IL-8, TNF-α, MCP-1, MIP-1β, PlGF, and VEGF-A, as well as aqueous IL-6, IL-8, PlGF, and VEGF-C. The levels of IL-8, PlGF, and VEGF-A in the vitreous and aqueous humor were found to be significantly associated with patients with PDR, while plasma cytokines did not show any correlation with these ocular fluids. In patients with PDR, IL-8, VEGF-A, and PlGF demonstrated a strong correlation between vitreous and aqueous humor, suggesting that aqueous humor can act as an analog for vitreous humor in studying some cytokines related to PDR [14]. The pilot study conducted by Bonfiglio et al. [13] investigated clinical outcomes and serum cytokine levels across six cohorts of participants. The findings revealed that there was no significant difference in PlGF serum levels between diabetic patients and control subjects; rather, these levels increased in NPDR patients one week following aflibercept treatment. This finding supports the argument, as described in previous studies, that the elevation of serum PlGF serves as a counter-regulatory mechanism, caused by the inhibition of VEGFR2 signaling by anti-VEGF agents or VEGFR tyrosine kinase inhibitors. There was no correlation between PlGF serum levels and either DR staging or clinical outcomes [13]. These findings were similar to the ones reported concerning the associations between serum and vitreous VEGF-A and between serum and vitreous VEGF-B and suggest an intravitreal synthesis of PlGF [21,22]. Considering that, intravitreal administration of a drug may have limited systemic effects [3]. Moreover, there was no significant association found between PlGF levels and CRT nor with MV. As a result, it was not possible to draw any conclusions from the available data. It is crucial to remember that obtaining a reliable correlation with a sample size of 21 diabetics would be difficult. Therefore, this question should be addressed in further research in a larger cohort of DR patients.

According to our results and other studies, the use of anti-PlGF could be an alternative to the current treatments. Treatment with anti-angiogenic agents for ocular pathologies arose a few years ago, initially with pegaptanib, then bevacizumab (off-label), followed by ranibizumab, aflibercept, and finally with faricimab intravitreal injections [23]. These anti-angiogenic therapies rapidly became the gold standard for the treatment of neovascular eye diseases [23]. There is no doubt that anti-angiogenic therapy is a hallmark in the history of the treatment of DR. However, it is important to improve outcomes in the treatment of these ocular diseases by minimizing acquired resistance to anti-VEF therapy and associated toxicity, particularly due to the lack of long-term safety data on VEGF inhibition and potential side effects. Furthermore, not all patients obtain a satisfactory response to its treatment, so it is crucial to continue the search for new molecular targets, therapeutic agents, and therapeutic strategies. Considering this, the inhibition of PlGF is a possible alternative to DR treatment since it regulates angiogenesis and vascular permeability in pathological conditions, and, thus, the inhibition of PlGF could minimize diabetic complications [24]. It must be emphasized that the under- or overexpression of PlGF did not affect normal vascular development or function, suggesting that the conceptualization of an anti-PlGF therapy could be safer than other anti-angiogenic molecules. However, regarding the role of PlGF in pathological neovascularization in cancer, Sheibani observed that the inhibitory activity of PlGF may be tumor-specific and not all anti-PlGF have antagonist activity [9]. If this observation may be extended to ocular diseases, it suggests that not all anti-PlGF treatments may be universally effective.

Notwithstanding, the efficiency of anti-PlGF drugs for the treatment of cancer and ocular diseases has been tested in several clinical trials. Van de Veire et al. demonstrated in an animal model that the monoclonal antibody 5D11D4 inhibits choroidal neovascularization, ocular angiogenesis, and inflammation by blocking PlGF [10]. TB-403 (THR 317) is another monoclonal antibody that binds to PlGF, blocking its interaction with VEGFR-1. The results of the phase I clinical trials demonstrated that TB403 was well-tolerated without increased risk of adverse effects in both healthy volunteers and terminally ill cancer patients [7,10]. In DME, two major phase II studies were performed: the first was a dose-finding study with 4 and 8 mg of THR 317, and the second was a comparison between THR 317 and ranibizumab (ClinicalTrials.gov Identifiers NCT03499223 and NCT03071068, respectively). The dose-finding study met the primary endpoint of safety for both the 4 mg and 8 mg doses. Regarding the second one, although the study showed that THR-317 and ranibizumab together are safe and well-tolerated, the comparison between THR-317 and ranibizumab showed no improvement at month three in the overall population.

Some authors suggest that a combined VEGF-A and PlGF inhibition resulted in a more effective reduction in vascular leakage and neovascularization than either agent alone, highlighting the synergistic potential of these two molecules [25]. Kowalczuk et al. [26] investigated the pro-angiogenic activity of PlGF on patients with DR, evaluating the effect of continuous over-expression of PlGF in the ocular media of rats through ciliary muscle electrotransfer. The findings showed that the continuous release of PlGF leads to vascular and retinal alterations that resemble the early manifestations of DR. PlGF and its receptor Flt-1 may be considered a potential regulatory target at this stage of the disease. Moreover, pathological conditions lead to direct effects of PlGF on endothelial migration through Flt-1, vascular permeabilization, and indirect effects on angiogenesis through Flk-1. When excessively produced in cells that produce VEGF, the VEGF/PlGF heterodimers induce both suppression and amplification of the pro-angiogenic effects of VEGF by disrupting the binding of Flk-1. Furthermore, this study demonstrated that PlGF may play a role in the initial phases of DR, particularly when only microaneurysms are present. The authors propose that PlGF may have a synergistic effect with VEGF during the initial phases of DR. Consequently, PlGF might be useful in mitigating the initial vascular abnormalities that occur during these stages of DR [26]. Long-term anti-VEGF-A suppression appears to induce atrophy of retinal pigment epithelium (RPE), triggers apoptosis, and heightens cellular vulnerability to oxidative stress [27], which are now recognized as key pathogenic events in vitreoretinal disorders [1,28]. On the other hand, PlGF plays a protective role for RPE cells, shielding them from apoptosis induced by serum starvation and maintaining the stability of VEGFR-2 in RPE [29]. Knocking down PlGF leads to VEGFR-2 protein instability, disrupting the signal transmission of the VEGFA/VEGFR-2 pathway and diminishing the protective effect of VEGF-A in RPE cells. Consequently, long-term VEGF-A inhibition in patients with neovascular age-related macular degeneration (AMD) may contribute to macular atrophy and could impact PlGF [29]. On the other hand, several implied that PlGF plays a role in subretinal fibrosis and that anti-PlGF can help ameliorate the associated symptoms. Klaassen et al. found that the intravitreal levels of PlGF and other pro-angiogenic mediators, such as PDGF and Ang-2, were strongly correlated to the degree of fibrosis in PDR [30]. Zhang et al. demonstrated that PlGF, mainly expressed in the RPE, is upregulated at the lesion site of subretinal fibrosis [31]. Nevertheless, the intravitreal injection of neutralizing antibodies targeting PlGF in RPE cells significantly inhibited the degree of subretinal fibrosis in choroidal neovascularization mice [31]. Therefore, the adjuvant treatment with anti-PlGF drugs could help overcome the lack of efficacy of many anti-VEGF compounds in (preventing) fibrosis, which can be explained by a concomitant upregulation of PlGF [7].

In our study, PlGF was found to be increased in the vitreous of DR patients, with higher levels observed in PDR patients compared to healthy controls without diabetes. Furthermore, we have noted an increase in PDR vitreous PlGF levels in comparison to NPDR, suggesting a potential increase in PlGF levels as the disease progresses. However, it is important to note that we are unable to establish this correlation conclusively due to the limited number of NPDR patients in our sample. In this phase of DR, PlGF inhibition may be beneficial in patients with retinal conditions associated with wound healing responses in reducing the process of fibrovascular scar formation, a common complication of VEGF inhibition [31]. Targeting PlGF should offer an additional treatment strategy for ocular pathologies with a neovascular component, but not all anti-PlGF antibodies are functional and demonstrate antagonistic activity [6].

The combined administration of anti-PlGF and anti-VEGFR-2 antibodies proved to induce a significant synergistic effect with a more than fourfold inhibition of neovascularization when compared to VEGFR-2 monotherapy [10]. The efficacy of combined PlGF/VEGF-A neutralization can be explained because these growth factors activate different signaling pathways upon receptor binding, inducing enhanced anti-angiogenic efficacy. However, in clinical practice, aflibercept that inhibits both VEGF-A and PlGF has not demonstrated superior efficacy to ranibizumab at two years in protocol T [32]. The recent approach based on gene therapy, a specific and targeted treatment with the potential for a sustained duration of therapeutic effect, represents another promising strategy. The work of Araújo et al. demonstrated that non-viral systems can effectively induce a sustained increase in the PEDF:PlGF ratio in the retina of mice under pathological conditions, creating a non-viral system in a pEPito-based vector capable of overexpressing PEDF to inhibit angiogenesis while suppressing PlGF [33].

In contrast to the essential role of VEGF-A in physiological and pathological angiogenesis and vasculogenesis, the role of PlGF in these events is restricted to pathological conditions, being considered for this reason a specific target for therapy. Considering all the evidence presented here, PlGF may represent an alternative or adjuvant target for the inhibition of angiogenesis, which could reinforce the effect of anti-VEGF drugs. However, this study has a few limitations. Firstly, the number of patients diagnosed with NPDR is small, as these patients rarely undergo vitrectomy. The number was further reduced to prevent bias since it was necessary to remove several patients who previously received anti-VEGF drugs, particularly aflibercept. Furthermore, all patients with additional systemic disorders that could affect the systemic levels of PlGF, such as oncological diseases, were excluded.

## Conclusions

This study found a significant increase in vitreous PIGF concentration among DR patients compared to a control group of non-diabetic individuals, suggesting its involvement in the pathogenesis of PDR. No significant association was found between CRT and PIGF, as well as between MV and PIGF, making it difficult to draw definitive conclusions from the available data. Moreover, in our study, no correlation was found between the concentrations of PlGF in vitreous and serum. The treatment for neovascular eye diseases has evolved with the introduction of anti-VEGF drugs; however, the efficacy of these drugs in inhibiting VEGF and angiogenesis remains debated. To improve outcomes, avoid resistance, and minimize toxicity, additional therapeutic agents should be investigated. PIGF, a protein that regulates angiogenesis and vascular permeability, emerges as a potential intervention. Furthermore, PIGF mediates both neovascularization and inflammation, and its role is restricted to pathological conditions. Clinical trials are needed to assess and validate the efficacy and safety of monoclonal antibodies targeting PIGF in the treatment of ocular pathologies, either in combination with anti-VEGF therapy or as a partial replacement of anti-VEGF drugs.
